# Estradiol and memory circuitry in the postmenopause: Modification by APOE4

**DOI:** 10.21203/rs.3.rs-10057599/v1

**Published:** 2026-08-04

**Authors:** Rahel A Schroeder, Rebecca C. Thurston, Minjie Wu, Howard J Aizenstein, Thomas K Karikari, M. Ilyas Kamboh, Ann D Cohen, Pauline M Maki

**Affiliations:** (1) Department of Psychology, University of Illinois Chicago, Chicago, IL, USA; (2) Department of Psychiatry, School of Medicine, University of Pittsburgh, Pittsburgh, PA, USA; (3) Departments of Psychiatry, Psychology, Epidemiology, and Clinical and Translational Science, University of Pittsburgh School of Medicine, Pittsburgh, PA, USA; (4) University of Pittsburgh Alzheimer’s Disease Research Center (ADRC), Pittsburgh, PA, USA; (5) Departments of Psychiatry, Psychology, and Obstetrics & Gynecology, University of Illinois Chicago, Chicago, IL, USA

**Keywords:** Alzheimer’s disease, estradiol, APOE4, fMRI, menopause, memory

## Abstract

**Background:**

Menopause-related declines in endogenous estrogen have been implicated in the higher risk of Alzheimer’s disease (AD) in female *APOE4* carriers compared to male *APOE4* carriers, but evidence remains inconclusive. Here we examined *APOE4*-related differences in the association of endogenous estradiol with patterns of hippocampal functional connectivity (HFC) during memory tasks, and how those patterns relate to AD biomarkers and memory performance.

**Methods:**

172 postmenopausal women (mean age=59.3±3.9 years, 83% white, 23.33% *APOE4* carriers) enrolled in MsBrain completed functional magnetic resonance imaging (fMRI) during a word memory task comprising encoding and recognition phases. Left and right HFC were determined by generalized psychophysiological interaction (gPPI) analysis. Interactive associations of estradiol levels and *APOE4* carrier status (*APOE3* homozygotes versus *APOE4* carriers) with whole-brain left and right HFC were analyzed via linear regression. In subsequent regression analyses, patterns of HFC were examined in relation to blood-based AD biomarkers, cognition and affect, stratifying by *APOE4* carrier status. Models controlled for age, education, and body mass index, and were cluster-corrected at p<.05.

**Results:**

*APOE4* status modified estradiol-related HFC to multiple brain areas, primarily frontal and temporal regions. For the encoding phase, higher estradiol levels were associated with lower left HFC and higher right HFC in *APOE4 carriers*, but with higher/neutral left HFC and lower/neutral right HFC in *APOE4* non-carriers. For the recognition phase, estradiol was related to higher right and left HFC in *APOE4* carriers and neutral/lower HFC in *APOE4* non-carriers. Estradiol-related HFC in midlife women was more strongly related to AD pathology in *APOE4* carriers versus *APOE4* non-carriers, and it was related to better memory performance only in non-carriers.

**Conclusion:**

In women *APOE4* carriers, higher estradiol was associated with a maladaptive pattern of HFC, which was in turn associated with an adverse AD risk profile as measured by AD biomarkers. Conversely, in *APOE4* non-carriers, endogenous estradiol appears to have a distinct, beneficial, effect on HFC. These results suggest that higher endogenous levels of estradiol associate with an early AD-related decompensation in memory circuitry in midlife women who carry a genetic risk for AD but a more favorable pattern in women without that genetic risk.

## Introduction

Women bear a disproportionate burden of Alzheimer’s disease (AD): of the 7.2 million Americans aged 65 years or older living with AD dementia, two-thirds are women (Alzheimer’s Association, 2025). Midlife changes in sex steroid hormones, namely estrogen declines during the menopause transition, have emerged as a plausible contributor to this sex difference (Mielke et al., 2014; Nebel et al., 2018), yet the mechanisms linking estrogen decline to AD risk in women remain poorly understood.

The perimenopause transition, which includes wide variability in sex steroid hormones and is characterized by changes in menstrual bleeding, is followed by the postmenopause, marked by 12 months without menstrual bleeding (Harlow et al., 2012; Tepper et al., 2012). In the postmenopause, which encompasses the remaining lifespan, levels of endogenous estradiol are at the lowest point in the lifespan and remain stable. However, there are considerable differences in estradiol levels between postmenopausal women (Tepper et al., 2012). Even at these physiologically low levels, endogenous estradiol continues to influence brain function, memory, and affect in beneficial ways (Schroeder et al., 2024). Longitudinal studies of cognitive performance reliably report declines in verbal memory over the menopause transition (Epperson et al., 2013; Greendale et al., 2009; Kilpi et al., 2020; Maki et al., 2021; Weber et al., 2021), consistent with extensive animal and human evidence for a role of estrogens in memory systems (Bailey et al., 2011; Jacobs et al., 2017; Maki et al., 2011; Ottowitz et al., 2008; Rapp et al., 2003; Wang et al., 2010; Woolley et al., 1990, 1996). What remains unclear is whether endogenous estradiol continues to shape memory circuitry in late midlife, after the menopause transition, and critically, whether such effects are modified by genetic risk for AD (i.e., *APOE4* genotype).

A key candidate mechanism is hippocampal functional connectivity (HFC), the coordinate activity between the hippocampus and distributed cortical regions that support learning, memory, and other cognitive tasks (Eichenbaum, 2017; Ranganath & Ritchey, 2012; Soltani Zangbar et al., 2020). HFC declines with age more rapidly in women than men (Ballard et al., 2023), tracks fluctuations in estradiol levels across the menstrual cycle in both humans and in rodents (Pritschet et al., 2020; Schoepfer et al., 2020), and improves following intravenous infusion of estradiol in postmenopausal women (Ottowitz et al., 2008). In those with AD, age-related HFC declines are more pronounced (Dautricourt et al., 2021), underscoring its relevance as an early marker of neurobiological vulnerability.

The picture is further complicated by *APOE4* genotype, the strongest genetic risk factor for AD, and whose effects are more pronounced in women than in men (Narasimhan et al., 2024; Neu et al., 2017). Exogenous sources of estrogen through menopausal hormone therapy (MHT) has shown beneficial effects on AD biomarkers in some (Depypere et al., 2023; Kantarci et al., 2016), but not all (Shadyab et al., 2026), studies. Critically, one observational study found that women carrying the *APOE4* allele using MHT exhibited significantly worse AD biomarker profiles compared to *APOE4* non-carriers (Jauregi-Zinkunegi et al., 2025). These associations highlight the modifying role of *APOE* genotype on the relationship between endogenous estradiol and memory circuitry.

In a large sample of late midlife, postmenopausal women, we investigated whether *APOE4* carrier status modifies the association between endogenous estradiol and HFC during verbal memory encoding and recognition. Having identified estradiol-related HFCs that differed by *APOE4* carrier status, we then tested whether those connections were adaptive by evaluating their associations with blood-based AD biomarkers and cognitive performance. Results from this study offers the first neuroimaging evidence of how genetic risk for AD reshaped the role of estrogen in memory circuitry, with direct implications for identifying early intervention targets to optimize brain health in women most at risk.

## Methods

### Sample

Participants in this study were enrolled in MsBrain (2017-2020), a cohort study of menopause and brain health conducted among community-dwelling women from the Pittsburgh, PA community (Thurston et al., 2016). Briefly, MsBrain exclusion criteria included: hysterectomy and/or bilateral oophorectomy; current pregnancy; and use of HT (oral or transdermal estrogen and/or progesterone), selective ER modulators, aromatase inhibitors, selective serotonin reuptake inhibitors or serotonin norepinephrine reuptake inhibitors, a reported history of dementia; seizure disorder; brain tumor; Parkinson disease; a history of head trauma with loss of consciousness; contraindications to MRI (e.g., metal in the body); current chemotherapy; and active substance use. For more detailed information regarding inclusion and exclusion criteria, refer to (Schroeder et al., 2024). The University of Pittsburgh Human Research Protection Office approved the research procedures, and all participants provided written, informed consent.

In total, 237 women completed neuroimaging (mean age 59.13 years +/− 4.26; 70.46% white; 14.77% high school education, 84.81% some college or higher). Of those women, nine were excluded due to incidental brain findings (e.g., stroke, tumor), six had missing or incomplete fMRI task data, five had excessive motion, seven had task behavior performance below chance (indicative of a lack of engagement in the task), four learned English as a second language, four were missing endogenous estrogen levels, two were perimenopausal defined by reported bleeding patterns as assessed by the STRAW+10 (Harlow et al., 2012) and ReSTAGE (Harlow et al., 2008) criteria, one had premature ovarian failure, 13 were past or present users of MHT, seven did not have *APOE* genotype data, and seven *APOE2* carriers were excluded. The final sample included 172 postmenopausal women.

### Measures

Verbal memory was assessed using the California Verbal Learning Test (CVLT)(Delis et al., 2002). Global cognitive ability was assessed via the Montreal Cognitive Assessment (MOCA)(Nasreddine et al., 2005). Phlebotomy was performed after a 12-hour overnight fast. Serum estradiol was assessed LC-MS/MS at the University of Pittsburgh’s Small Biomarker Core, with inter- and intra-assay coefficients of variation of 8.1% and a lower limit of detection of 1.0 pg/ml(Nelson et al., 2004). *APOE* genotype was performed at the Neurogenetics Core at the University of Pittsburgh Alzheimer’s Disease Research Center. Two SNPs were converted into the conventional six *APOE* genotypes (2/2, 2/3, 3/3, 3/4, 2/4, and 4/4). After excluding *APOE2* carriers, genotypes were divided into groups: *APOE4* carriers (3/4 heterozygotes and 4/4 homozygotes) and *APOE4* non-carriers (3/3 homozygotes). Blood-based AD biomarker concentrations were measured using Single molecule array (SIMOA) technology on an HD-X instrument (Quanterix, Billerica, MA, USA). Plasma Aβ42 and Aβ40 were measured with the Neurology 4-Plex E (#103670) commercial assays from Quanterix (Billerica, MA, USA) (Keshavan et al., 2021). Plasma pTau 181 and pTau 231 were measured using validated in-house assays following published protocols (Ashton et al., 2021; Karikari et al., 2021). For each assay, two or three quality control samples of different concentrations were analyzed in duplicates both at the start and the end of each technical run to estimate reproducibility. The pooled quality control data showed that the within- and between-run variations in signal were <20% (mostly around 10%). More information regarding the neuropsychological testing, phlebotomy methods, and a detailed description of the neuroimaging protocol and verbal memory fMRI task can be found in prior work (Schroeder et al., 2024). Briefly, participants underwent neuroimaging at the MR Research Center of the University of Pittsburgh on a 3T Siemens Tim Trio MR scanner, with a Siemens 64-channel head coil. Structural data were acquired using a T1-weighted 3-dimensional magnetization-prepared rapid gradient echo (MPRAGE: TR/TI/FA = 2300/900/9°, voxel size = 1 mm*1 mm*1 mm, Grappa 2). Whole-brain fMRI data were acquired axially (parallel to anterior commissure—posterior commissure line) using gradient-echo echo-planar imaging (EPI) sequence: repetition time (TR) = 1 seconds, echo time (TE) = 30 ms, flip angle = 45°, FOV = 220 × 220 mm2, acquisition matrix 96× 96, slice thickness/gap = 2.3/0 mm (voxel size = 2.3× 2.3× 2.3 mm3), 60 axial slices. T1-weighted MPRAGE images were used for normalization of fMRI data into the Montreal Neurological Institute (MNI) template. The fMRI task comprised of a word encoding phase and a word recognition phase separated by a 15-minute delay, modeled after the Hopkins Verbal Learning Test (HVLT)(Brandt & Benedict, 2001) and the CVLT (Delis et al., 2002). Each phase of the task, encoding and recognition, were comprised of 10 blocks, with 5 experimental blocks of 6 words each alternating with 5 control blocks of 6 words each. The primary outcomes were the number of words correctly recognized during the experimental blocks and the number of correct rejections of unrelated and related distractors.

## Analysis

Predictors were assessed for outliers and normality. Sixteen women had estradiol levels below the LC-MS/MS level of detection (LOD) of 1 pg/mL and were assigned the value of the lowest LOD for estradiol LC-MS/MS of 1. Estradiol levels and BMI were log-transformed for normality.

Left and right hippocampal seeds based on the automated anatomic labeling atlas were used to estimate functional connectivity between the hippocampus and voxels in the brain using Generalized psychophysiological interaction (gPPI) analysis (Friston et al., 1997; Tzourio-Mazoyer et al., 2002). Principal time series (i.e., the eigenvariate) were generated for each seed region, left and right hippocampus, using singular value decomposition (in MATLAB 2020) from hippocampal fMRI data during the verbal encoding task. Principal time series of the seed region (left or right hippocampus), task conditions (experimental and control blocks), interaction variables (seed times series * task condition), as well as motion parameters were included in the design matrix. Motion parameters [R R^2^ R_t-1_ R_t-1_^2^] (R representing motion estimates [x y z pitch yaw roll]) and motion spikes (detected based on framewise displacement [FD] < 0.5 mm) were included as confounding regressors to control for artifacts induced by in-scanner head motion. Connectivity maps (left or right hippocampus) during the encoding and recognition tasks were computed for each participant. Whole brain results were restricted to clusters composed of at least 50% grey matter. To control for multiple comparisons, joint height and extent thresholds were determined via Monte Carlo simulations (10,000 iterations) with an a priori whole-brain grey matter mask (AlphaSim, AFNI) for a corrected p < 0.05 (Cox, 1996). Due to the known effects of age and education on cognitive ability (Ardila et al., 2000; Deary et al., 2009), and BMI on estradiol (Tepper et al., 2012), each were entered as covariates in the models. All post hoc associations were analyzed using linear regression, lme4 Package (Bates et al., 2015) in R (R Development Core Team, 2018).

A linear regression model was used to test interactive associations between two independent measures - continuous levels of endogenous estradiol levels and binary *APOE4* carrier status (*APOE4* carriers versus non-carriers) – with the dependent measure of the magnitude of HFC to regions of the whole brain, controlling for covariates of age, years of education and BMI. Although African ancestry is associated with greater frequency of the APOE4 allele compared to European ancestry (Phan et al., 2020), in this study race was not statistically significantly associated with APOE4 frequency (*r*=.01, *p*=.86) and was therefor not included as a covariate in the models. Separate models were used for the left and right hippocampal seed regions.

In post-hoc models to explore interpretations of the HFC associations, we tested associations between the magnitude of HFC to regions of the whole brain with blood-based AD biomarkers – continuous measures of the levels of tau and amyloid-beta (pTau 181, pTau 231, and Aβ42/40) – controlling for covariates of age, years of education, and BMI. Models were stratified by *APOE4* carrier status. Separate linear regression models were run for the tau and Aβ measures.

Finally, in post-hoc exploratory models we tested associations between the magnitude of HFC to regions of the whole brain with the dependent measures of memory performance–continuous measures of CVLT measures (CVLT total learning, CVLT semantic clustering, short delay recall, and long delay recall), task behavioral measures (task accuracy, task verbal learning, and task semantic clustering), and global cognition (MOCA) – controlling for covariates of age, years of education and BMI. Models were stratified by *APOE4* carrier status.

## Results

The sample comprised late midlife women (mean age 59.38 +/− 3.96 years), all of whom were postmenopausal, 83.14% of whom were white, average years of education 15.71 and 23.33% were *APOE4* carriers (3 *APOE* 4/4 homozygotes and 37 *APOE* 3/4 heterozygotes along with 125 *APOE* 3/3 homozygotes ). See Table 1. *APOE4* carrier status was not significantly associated with levels of endogenous estradiol (β[SE]=0.058[0.054], p=0.286), controlling for age, years of education, and BMI.

### Interactive association of endogenous estradiol levels with HFC by *APOE4* carrier status Encoding.

**Figure 1.**
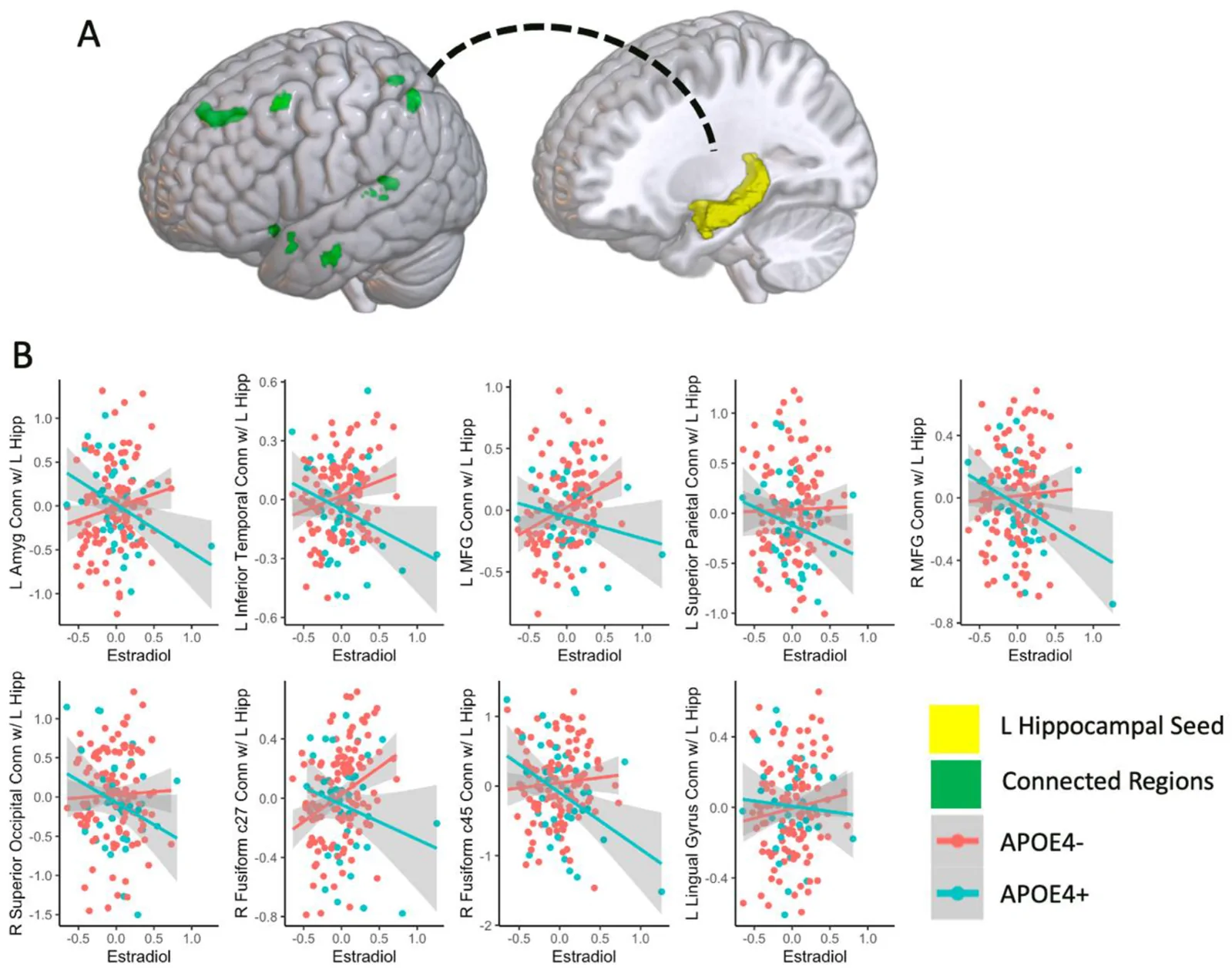
Associations of endogenous estradiol with left hippocampal functional connections during encoding that vary by *APOE4* carrier status. A) Location of anatomically defined mask of the left hippocampus seed region (yellow) and brain regions where functional connectivity to the left hippocampus (green) was associated with endogenous estradiol differently in *APOE4* carriers (blue) versus *APOE4* non-carriers (red). B) Scatterplot of the association of endogenous estradiol with the magnitude of functional connectivity by *APOE4* status. These associations were generally negative in *APOE4* carriers but positive or neutral in *APOE4* non-carriers women. Parameters are z-scored residuals from regressions controlling for age, years of education, and BMI. Abbreviations: (L) left, (R) right hemisphere, (c) cluster size for regions with multiple significant areas, (Conn w/) connectivity with, (Hipp) hippocampus, (*APOE4*−) *APOE4* non-carriers, (*APOE4*+) *APOE4* carriers. Note: All fMRI results are cluster-size corrected at p<.05. Error bars are 95% confidence intervals.

**Figure 2.**
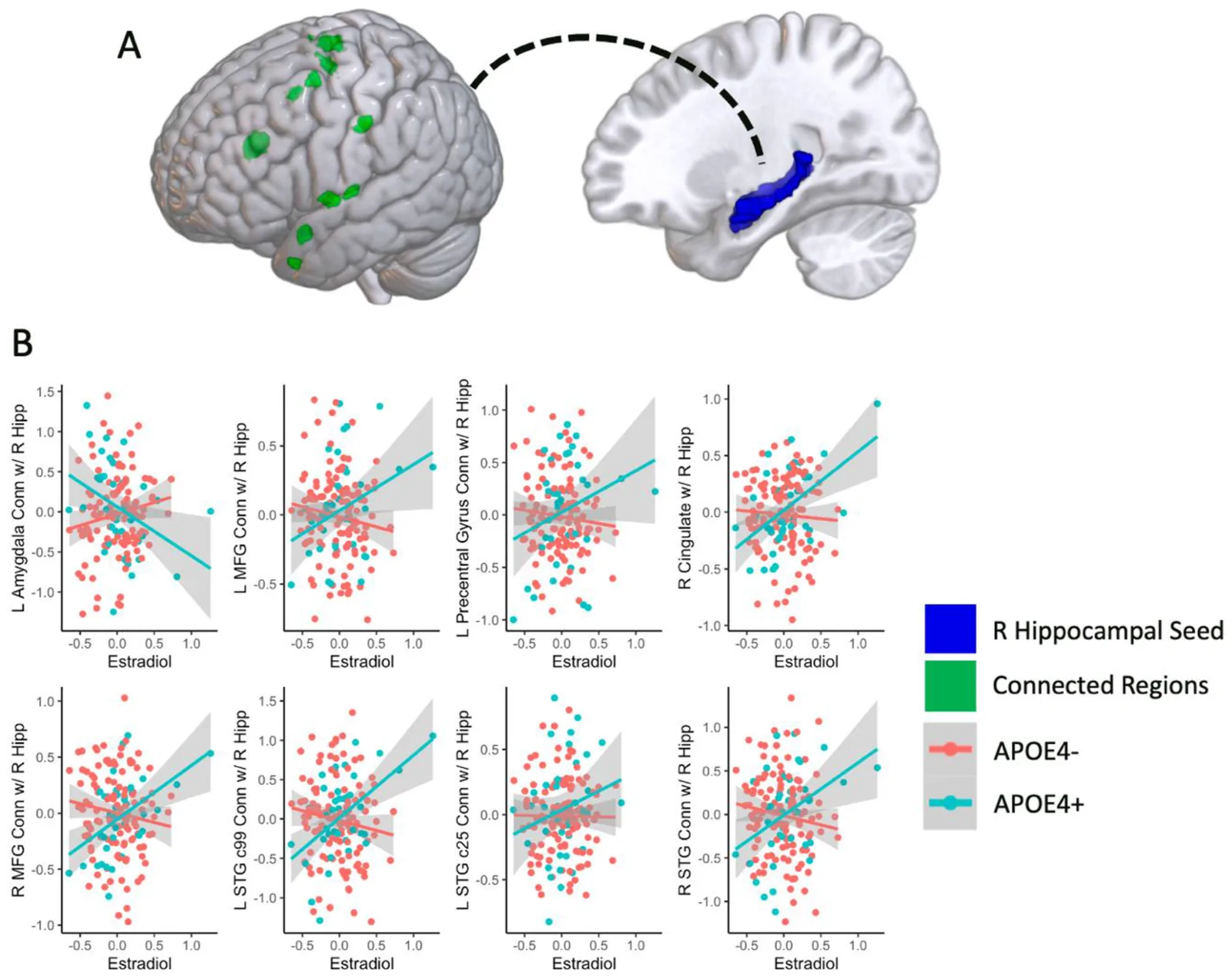
Associations of endogenous estradiol with right hippocampal functional connections during encoding that vary by *APOE4* carrier status. A) Location of anatomically defined mask of the right hippocampus seed region (dark blue) and brain regions where functional connectivity to the left hippocampus (green) was associated with endogenous estradiol differently in *APOE4* carriers (blue) versus *APOE4* non-carriers (red). B) Scatterplot of the association of endogenous estradiol with the magnitude of functional connectivity by *APOE4* status. These associations were generally positive in *APOE4* carriers but negative or neutral in *APOE4* non-carriers. Parameters are z-scored residuals from regressions controlling for age, years of education, and BMI. Abbreviations: (L) left, (R) right hemisphere, (c) cluster size for regions with multiple significant areas, (Conn w/) connectivity with, (Hipp) hippocampus, (*APOE4*−) *APOE4* non-carriers, (*APOE4*+) *APOE4* carriers. Note: All fMRI results are cluster-size corrected at p<.05. Error bars are 95% confidence intervals.

Tables 2 and 4, Figures 1 and 2, show HFC in brain regions where the magnitude of functional connectivity varied by estradiol and *APOE4* carrier status during the encoding phase. During the encoding phase, *APOE4* carriers with higher levels of endogenous estradiol showed decreased functional connectivity of the left hippocampus to the left inferior temporal lobe (ITL), left superior parietal lobe (SPL), right middle frontal gyrus (MFG), right fusiform, left MFG, left amygdala/parahippocampal gyrus (PHG), left lingual gyrus, and right superior occipital lobe.

Specifically, in analyses stratified by *APOE4* status, during encoding, *APOE4* carriers with higher levels of endogenous estradiol had lower left hippocampus-to-left ITL, left hippocampus-to-right MFG, left hippocampus-to-right fusiform, left hippocampus-to-left MFG, and left hippocampus-to-left amygdala/PHG connectivity. *APOE4* carriers with higher levels of endogenous estradiol also had greater right hippocampus-to-right cingulate gyrus, right hippocampus-to-right MFG, right hippocampus-to-right STG, right hippocampus-to-left superior temporal gyrus (STG), and right hippocampus-to-left amygdala/PHG connectivity.

Among *APOE4* non-carriers, higher levels of endogenous estradiol was associated with greater connectivity between the left hippocampus-to-left ITL, left hippocampus-to-right MFG, and left hippocampus-to-right fusiform. *APOE4* non-carriers did not show significant associations between estradiol with right HFC during encoding.

#### Recognition.

**Figure 3.**
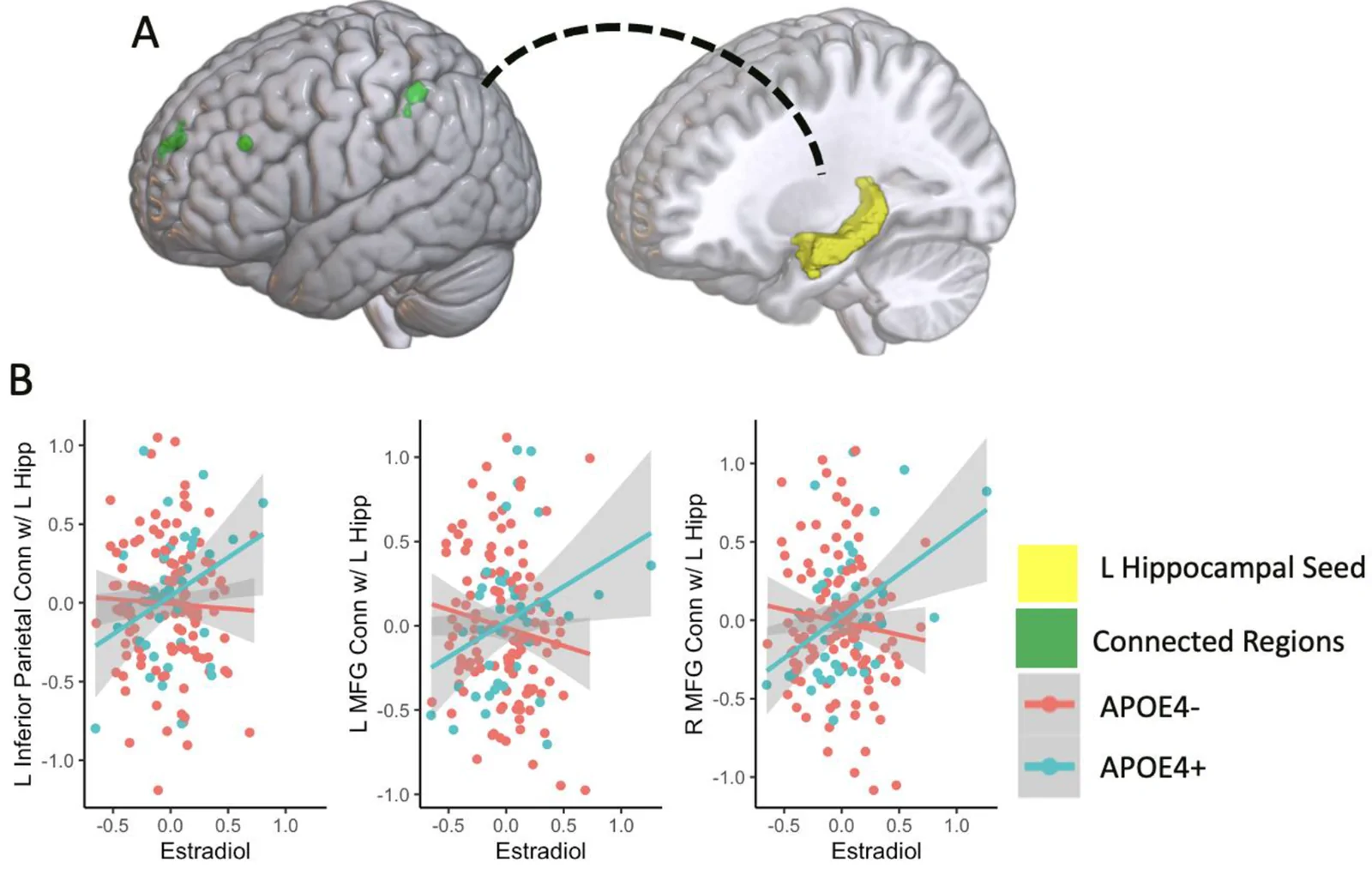
Associations of endogenous estradiol with left hippocampal functional connections during recognition that vary by *APOE4* carrier status. A) Location of anatomically defined mask of the left hippocampus seed region (yellow) and brain regions where functional connectivity to the left hippocampus (green) was associated with endogenous estradiol differently in *APOE4* carriers (blue) versus *APOE4* non-carriers (red). B) Scatterplot of the association of endogenous estradiol with the magnitude of functional connectivity by *APOE4* status. In general, these associations were positive in *APOE4* carriers neutral in *APOE4* non-carriers. Parameters are z-scored residuals from regressions controlling for age, years of education, and BMI. Abbreviations: (L) left, (R) right hemisphere, (c) cluster size for regions with multiple significant areas, (Conn w/) connectivity with, (Hipp) hippocampus, (*APOE4*−) *APOE4* non-carriers, (*APOE4*+) *APOE4* carriers. Note: All fMRI results are cluster-size corrected at p<.05. Error bars are 95% confidence intervals.

**Figure 4.**
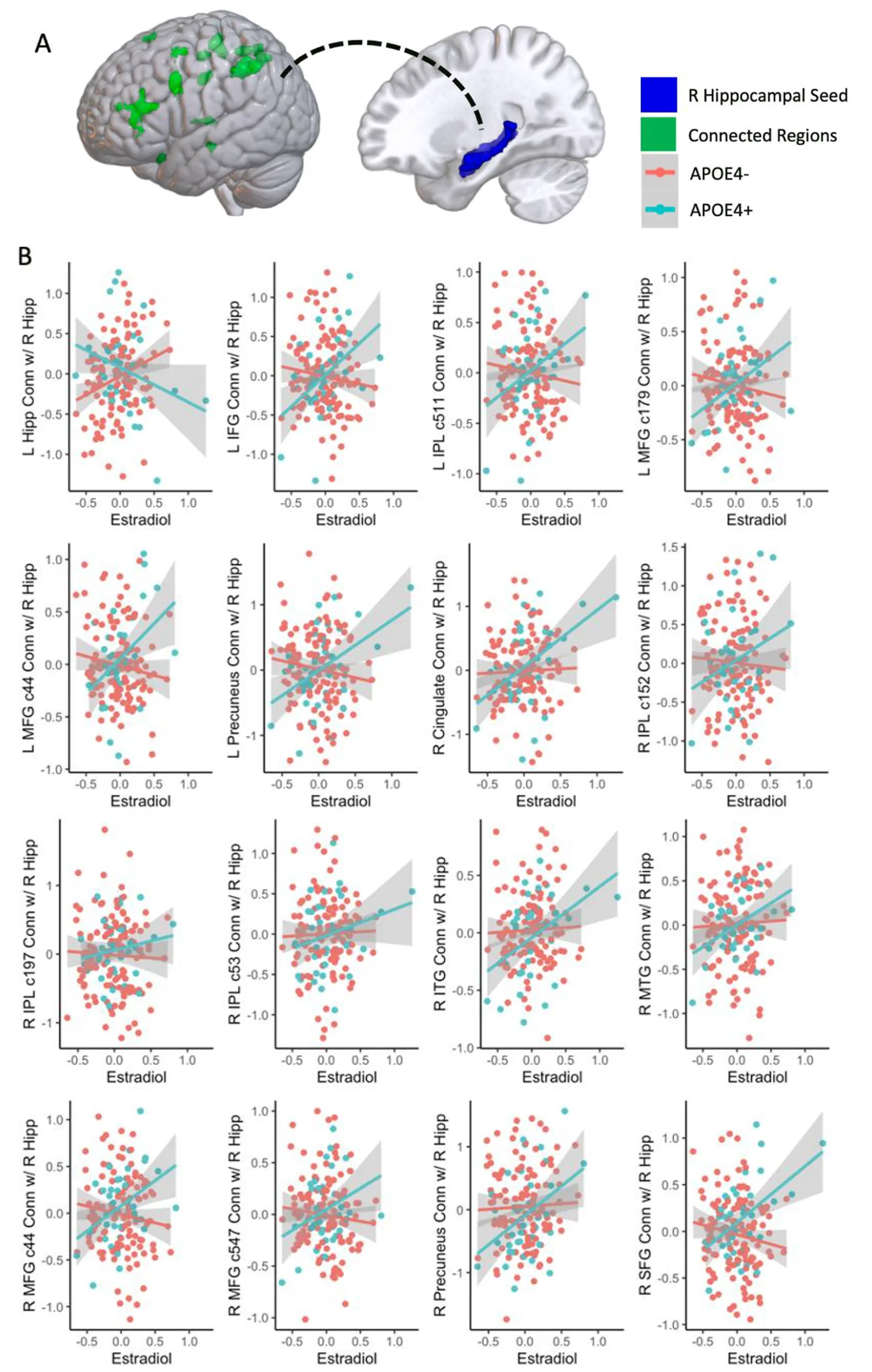
Associations of endogenous estradiol levels in right hippocampal functional connections during verbal recognition that vary by *APOE4* carrier status. A) Location of anatomically defined mask of the left hippocampus seed region (dark blue) and brain regions where functional connectivity to the left hippocampus (green) was associated with endogenous estradiol dfferently in *APOE4* carriers (blue) versus *APOE4* non-carriers (red). B) Scatterplot of the association of endogenous estradiol with the magnitude of functional connectivity by *APOE4* status. In general, these associations were positive in *APOE4* carriers but negative or neutral in *APOE4* non-carriers. Parameters are z-scored residuals from regressions controlling for age, years of education, and BMI. Abbreviations: (L) left, (R) right hemisphere, (c) cluster size for regions with multiple significant areas, (Conn w/) connectivity with, (Hipp) hippocampus, (*APOE4*−) *APOE4* non-carriers, (*APOE4*+) *APOE4* carriers. Note: All fMRI results are cluster-size corrected at p<.05. Error bars are 95% confidence intervals.

Tables 3 and 4, and Figures 3 and 4, show HFCs that varied by estradiol and *APOE4* carrier status during the recognition phase. During the recognition phase, *APOE4* carriers with higher levels of endogenous estradiol showed increased functional connectivity of the left hippocampus to the left inferior parietal lobe (IPL), right MFG, and left MFG. In the right hippocampus, *APOE4* carriers with higher levels of endogenous estradiol showed increased functional connectivity to the right MFG, left IPL, left inferior frontal gyrus (IFG), left MFG, right IPL, right superior frontal gyrus (SFG), right cingulate gyrus, right inferior temporal gyrus (ITG), right IPL, right MTG, right precuneus, left precuneus, and left hippocampus.

In *APOE*-stratified analyses of the recognition phase, among *APOE4* carriers, greater levels of endogenous estradiol were associated with increased connectivity with left hippocampus-to-left IPL, left hippocampus-to-right MFG, and left hippocampus-to-left MFG connectivity. In *APOE4* carriers, greater levels of endogenous estradiol were associated with increased right hippocampus-to-right MFG, right hippocampus-to-left IPL, right hippocampus-to-left IFG, right hippocampus-to-left MFG, right hippocampus-to-right IPL, right hippocampus-to-right MFG, right hippocampus-to-right SFG, right hippocampus-to-right cingulate gyrus, right hippocampus-to-right ITG, right hippocampus-to-right precuneus, and right hippocampus-to-left precuneus connectivity. In *APOE4* carriers, greater levels of endogenous estradiol were negatively associated with right hippocampus-to-left hippocampus connectivity. *APOE4* carriers did not show significant associations between endogenous estradiol and right hippocampus-to-right ITG or right IPL connectivity.

In *APOE*-stratified analyses of HFC in midlife women during the recognition phase, among *APOE4* non-carriers, greater levels of endogenous estradiol were associated with greater right hippocampus-to-left hippocampus connectivity. No associations with levels of endogenous estradiol and left HFC in *APOE4* non-carriers were seen during recognition.

### Post-Hoc models of blood-based AD biomarkers

#### Encoding.

Table 4, and Supplemental Tables 1 and 2, show associations of blood-based AD biomarkers with HFC during the encoding phase.

In stratified analyses during encoding, in *APOE4* carriers, increased left hippocampus-to-right fusiform connectivity was associated with greater AD pathology as shown by greater levels of pTau 181 and pTau 231, and lower levels of Aβ42/40 with left hippocampus-to-left amygdala/PHG connectivity. During encoding in *APOE4* carriers, increased right hippocampus-to-right cingulate gyrus connectivity was associated with greater AD pathology as shown by lower levels of Aβ42/40 and right hippocampus-to-right STG connectivity was associated with decreased AD pathology as shown by lower levels of pTau 181.

#### Recognition.

Table 4, and Supplemental Tables 3 and 4, show associations of blood-based AD biomarkers with HFC during the recognition phase.

In stratified analyses during recognition in *APOE4* carriers, greater AD pathology, as shown by lower levels of Aβ42/40, was found with increased right hippocampus-to-right IPL, right hippocampus-to-left MFG, and right hippocampus-to-right SFG connectivity.

In stratified analyses during recognition in *APOE4* non-carriers, right hippocampus-to-left IPL connectivity and right hippocampus-to-right precuneus and right hippocampus-to-left IPL connectivity were associated with increased AD pathology, as shown by greater levels of pTau 181.

Left HFC during recognition was not significantly associated with AD biomarkers in women *APOE4* carriers or *APOE4* non-carriers.

### Post-hoc models of cognition and affect

#### Encoding.

Table 4, and Supplemental Tables 5 and 6, show associations with measures of verbal memory performance and global cognition with HFC during the encoding phase.

In stratified analyses during encoding, in *APOE4* carriers, greater left hippocampus-to-left superior parietal connectivity was associated with a lower MOCA score indicating poorer global cognitive performance. Right HFC during encoding was not significantly associated with measures of cognition.

In stratified analyses during encoding in *APOE4* non-carriers, greater left hippocampus-to-right superior occipital connectivity was associated with better task verbal learning score. Increased right hippocampus-to-right cingulate gyrus connectivity was associated with better CVLT-assessed long delay recall performance. Increased right hippocampus-to-right MFG connectivity was associated with better CVLT-assessed semantic clustering, long delay recall, task verbal learning, and task semantic clustering. Increased right hippocampus-to-left MFG connectivity was associated with better task verbal learning. Increased right hippocampus-to-left STG connectivity was associated with better CVLT-assessed semantic clustering. Increased right hippocampus-to-right STG connectivity was associated with a higher MOCA score, and higher CVLT verbal learning, semantic clustering, short delay recall, long delay recall, task verbal learning, and task semantic clustering scores. Increased right hippocampus-to-left precentral gyrus connectivity was associated with better CVLT semantic clustering, task verbal learning, and task semantic clustering. Increased right hippocampus-to-left STG connectivity was associated with a higher MOCA score, task verbal learning, and task semantic clustering score.

#### Recognition.

Table 5, and Supplemental Tables 7 through 8, show associations with measures of verbal memory performance and global cognition with HFC during the recognition phase.

In stratified analyses during the recognition phase among *APOE4* carriers, greater left hippocampus-to-left IPL connectivity was associated with better short delay recall performance.

Increased right hippocampus-to-right SFG connectivity was associated with worse CVLT verbal learning and semantic clustering. Increased right hippocampus-to-right ITG connectivity was associated with better CVLT verbal learning. Increased right hippocampus-to-right MTG connectivity was associated with better MOCA score, task verbal learning, and task semantic clustering.

In stratified analyses during the recognition phase among *APOE4* non-carriers, increased right hippocampus-to-right SFG connectivity was associated with better MOCA score and task semantic clustering.

## Discussion

The goal of this study was to determine whether endogenous estradiol shapes memory circuitry differently depending on *APOE4* carrier status in late midlife, postmenopausal women, and whether any such differences carry clinical significance. Our findings reveal a striking divergence: in *APOE4* non-carriers, higher endogenous estradiol was associated with patterns of HFC that were beneficial for verbal memory performance, while in *APOE4* carriers, estradiol-related HFC patterns were not only cognitively inert but associated with unfavorable AD biomarker profiles. These results provide the first neuroimaging evidence that APOE4 carrier status fundamentally alters the relationship between endogenous estrogen and memory circuitry in postmenopausal women.

During encoding, *APOE4* carrier status modified estadiol-associated HFC across frontal, temporal, and limbic regions. These regions have a high density of estrogen receptors (Barth et al., 2015; Öterlund et al., 2000; Osterlund & Hurd, 2001) and are central to verbal memory in midlife women (Jacobs et al., 2017; Ottowitz et al., 2008; Schroeder et al., 2024). A particularly striking pattern emerge along hemispheric lines; in *APOE4* carriers, higher estradiol was associated with lower left HFC and higher right HFC, whereas in *APOE4* non-carriers opposite pattern was observed. This hemispheric difference was consistent across regions, with the notable exception of right HFC to the amygdala, where higher estradiol was associated with decreased connectivity among *APOE4* carriers. The consistency of this opposing laterality effect across multiple connections suggests a systematic difference, driven by *APOE* genotype modifying the influence of estradiol on memory networks.

The cognitive implications of these estradiol-associated encoding patterns were equally divergent by *APOE4* carrier status. Positive associations between estradiol-related HFC and memory performance were not seen in any region for the *APOE4* carriers. In *APOE4* non-carriers, right estradiol-related HFCs were broadly and positively associated with verbal memory performance as measured by the CVLT. To contextualize these findings, we compared the present results against normative HFC data from 171 women in the MsBrain cohort study (Wugalter et al., 2025), in which HFCs associated with better verbal memory performance on the CVLT were identified independent of estradiol or *APOE* genotype. Five of the estradiol- and *APOE4*-modified HFCs identified in the present study overlapped with that normative set. In *APOE4* non-carriers, these connections were positively related to both estradiol and memory, including the right hippocampal-to-right cingulate gyrus, two regions in the right hippocampus-to-left STG and the right hippocampal-to-right STG. In APOE4 carriers, the same connections were either unrelated or negatively related to memory performance, despite being among the strongest estradiol-associated HFCs observed (right HFC to the right cingulate gyrus and right STG). Further, left hippocampus-to-right MFG connectivity was associated with better verbal memory performance in the normative study, which was seen again in the present study among the *APOE4* non-carriers. Strikingly, this connectivity in *APOE4* carriers was negatively associated with estradiol and unrelated to CVLT performance. This pattern is most consistent with a robust but ultimately unsuccessful compensatory response where the circuitry is engaged, but the engagement does not translate into cognitive benefit.

The AD biomarker data reinforced this interpretation and revealed an additional layer of hemispheric complexity in *APOE4* carriers. Reduced left HFC associated with higher estradiol in *APOE4* carriers was accompanied by elevated pTau 181, elevated pTau 231, and lower Aβ42/40 — a convergent biomarker signature indicative of greater AD pathological burden. In contrast to the left, increased right HFC in *APOE4* carriers showed a more mixed picture: right HFC to the cingulate gyrus was associated with lower Aβ42/40 (unfavorable), while right HFC to the superior temporal gyrus was associated with lower pTau 181 (favorable), suggesting a potential partial and regionally specific compensation. In *APOE4* non-carriers, three left HFCs were associated with higher estradiol, one of which (left ITL), was associated with higher pTau 231 (unfavorable). Right HFC in APOE4 non-carriers to left inferior parietal lobe was associated with higher levels of pTau 181 (unfavorable). However, that association may have limited clinical significance since estradiol had only a small, nonsignificant association with this connection in *APOE4* non-carriers. Overall, the verbal encoding findings indicate that APOE4 carriers engage estradiol-related compensatory connectivity in right hippocampal networks that neither rescues cognitive performance nor fully overcomes underlying AD pathology.

During recognition, estradiol-related HFC again differed markedly by *APOE4* carrier status, with the predominant pattern being increased HFC in *APOE4* carriers and decreased or absent HFC associations in *APOE4* non-carriers. Right HFCs were larger and more numerous compared to left HFCs, with the largest functional connections observed from the right hippocampus to the right MFG/IFG (547 mm^3^) and right hippocampus to right IPL (511 mm^3^). Other HFCs included connections to the bilateral middle and inferior frontal gyri, right superior frontal gyrus, bilateral inferior parietal lobe, right cingulate gyrus, and bilateral precuneus. Among *APOE4* carriers, estradiol-related increases in right HFC to four regions (i.e. two clusters in left MFG, and one each in right IFL and right SFG) were associated with worse Aβ42/40, and right HFC to three regions (i.e. right HFC to both right SFG and right ITG) with poorer CVLT performance, further supporting the interpretation that heightened connectivity in this group reflects potentially maladaptive rather than compensatory activity. Notably, in *APOE4* non-carriers, right HFC to the inferior parietal lobe and precuneus during recognition was associated with elevated pTau 181 — a finding that warrants follow-up given the otherwise favorable profile in this group.

These findings extend a growing literature on the intersection of estrogen and *APOE4* in AD risk. While prior work has demonstrated a critical role for estradiol in HFC in postmenopausal women (Jacobs et al., 2017; Ottowitz et al., 2008; Schroeder et al., 2024) and *APOE4*-dependent effects of MHT on AD biomarkers (Jauregi-Zinkunegi et al., 2025), the present study is the first to characterize how *APOE4* genotype modifies the neurobiological effects of endogenous estradiol on task-based memory circuitry. This distinction is critical because effects of physiologically low endogenous estradiol cannot be assumed to mirror those of exogenously administered MHT, and the discrepant findings across MHT studies (beneficial in some: Depypere et al., 2023; Kantarci et al., 2016; neutral: Shadyab et al., 2026; or harmful in others: Jauregi-Zinkunegi et al., 2025) likely reflect this complexity. Our findings in postmenopausal women are also consistent with animal models showing that physiological estradiol fails to benefit, and may harm, *APOE4* transgenic mice, including estradiol-associated increases in Aβ (Kunzler et al., 2014) and declines in hippocampal memory and structural plasticity (Taxier et al., 2022).

This study has several strengths. This is the largest neuroimaging investigation of *APOE4* carrier status as a modifier of estradiol-associated HFC in postmenopausal. Estradiol was quantified using LC-MS/MS, the gold standard for ultra-low levels of endogenous estrogens in postmenopausal women (Nelson et al., 2004). The use of blood-based AD biomarkers in this area of research is novel (Ashton et al., 2021; Karikari et al., 2020, 2021; Keshavan et al., 2021). The generalizability of these findings is strong, with both race proportions and *APOE4* frequencies reflective of the U.S. population (Farrer et al., 1997; U.S. Census Bureau, 2021). A limitation is that the sample size of *APOE2* carriers was insufficient to evaluate the potentially protective role of the *APOE2* allele on HFC.

## Conclusion

This study provides the first neuroimaging evidence that *APOE4* carrier status fundamentally alters the relationship between endogenous estradiol and memory circuitry in postmenopausal women. While higher estradiol was associated with favorable hippocampal functional connectivity and better verbal memory performance in *APOE4* non-carriers, these benefits were absent in *APOE4* carriers, and were replaced instead by estradiol-related connectivity patterns linked to greater AD pathological burden. Broadly these findings suggest that higher functional decompensation in memory circuitry in *APOE4* carriers. These findings elucidate mechanisms during the menopause transition beyond estrogen loss, that may contribute to AD-related neurobiological vulnerability in women.

## Supplementary Material

1

## Figures and Tables

**Figure 5.**
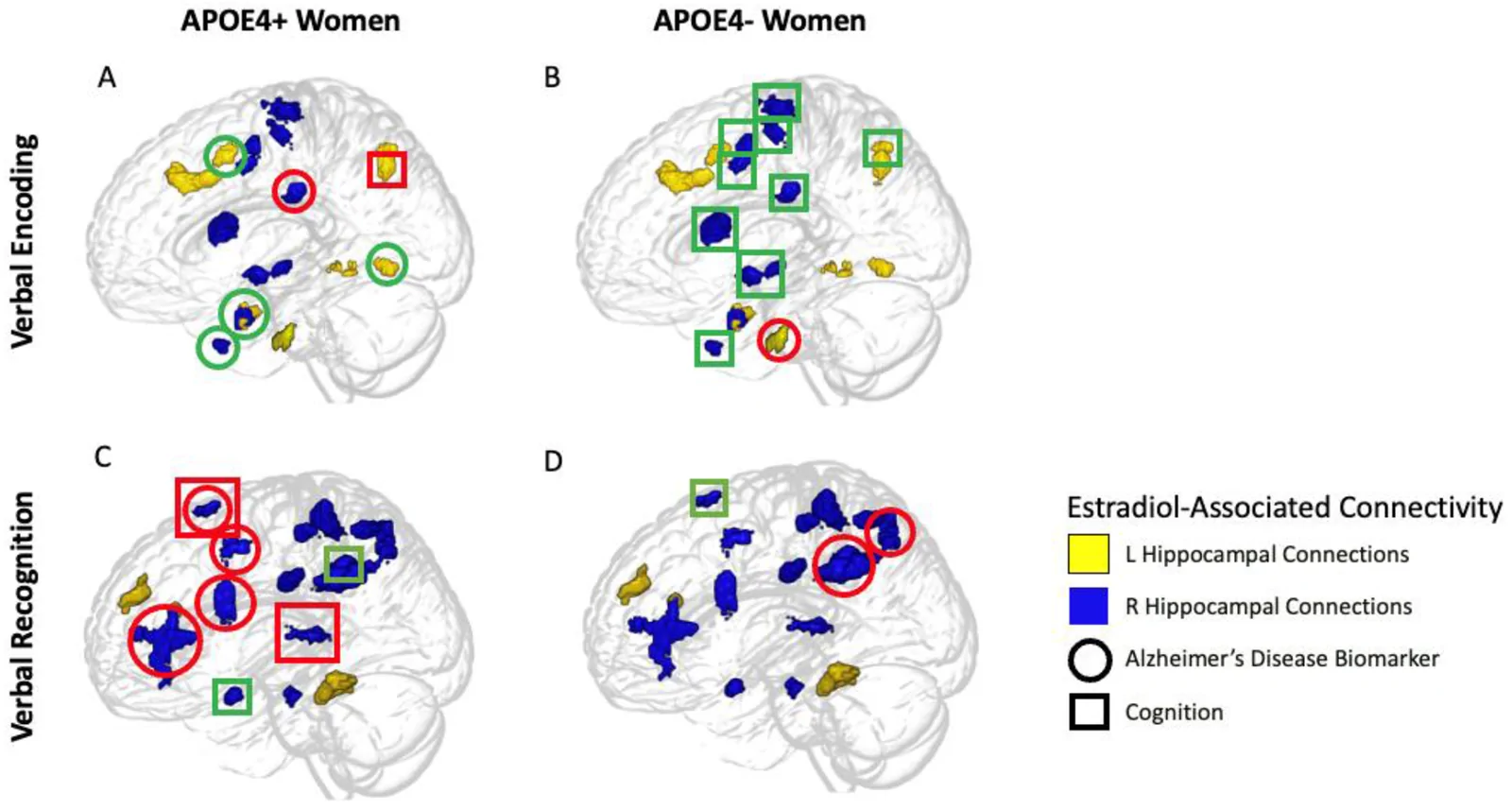
Summarization of associations with endogenous estradiol levels in hippocampal functional connections that vary by *APOE4* carrier status and influence of Alzheimer’s biomarkers and cognition. Brain maps show significant *APOE4*-related differences in estradiol-related hippocampal functional connectivity (HFC)(40 *APOE4* carriers, 132 *APOE4* non-carriers). Left HFCs are in yellow, right HFCs are in blue; regions surrounded by a circle were statistically associated with blood-based AD biomarkers and a square indicates regions statistically associated with cognition, where green indicates a beneficial association and red indicates a detrimental association. Panels A and B depict encoding results of associations between estradiol and HFC among *APOE4* carriers and *APOE4* non-carriers. Areas in yellow represent left HFC where, compared to non-carriers, *APOE4* carriers with higher levels of estradiol showed decreased functional connectivity. Areas in blue represent right HFCs, where compared to non-carriers, *APOE4* carriers with higher levels of estradiol showed increased functional connectivity (except for the left amygdala/parahippocampal gyrus where the *APOE4*-related differences were in the opposite direction). Panels C and D depict recognition results. Areas in yellow represent left HFC where, compared to *APOE4* non-carriers, *APOE4* carriers with higher levels of estradiol showed increased functional connectivity. Areas in blue represent right HFCs, where compared to non-carriers, *APOE4* carriers with higher levels of estradiol showed increased functional connectivity (except for the left hippocampus/parahippocampal gyrus where the *APOE4*-related differences were in the opposite direction).

**Table 1.** Participant Characteristics.

N	172
Age, M (Range, SD)	59.38 (49-67, 3.96)
Race/ethnicity, N (%)	
White	143 (83.14)
Black	23 (13.37)
Asian/Pacific Islander	2 (1.16)
Mixed Race	4 (2.33)
Education, M (SD)	15.71 (2.29)
*APOE4* Carrier, N (%)	40 (23.33)
Body mass index, M (SD)	28.39 (6.08)
Estradiol, pg/mL, Median (SD)	3.0 (4.96)
Estrone, pg/mL, Median (SD)	29.0 (17.02)
Depressive symptoms (CESD), Mean (SD)	7.26 (7.17)
Anxious symptoms (STAI), Mean (SD)	
State	30.40 (9.56)
Trait	31.68 (8.24)
Montreal Cognitive Assessment, Mean (SD)	26.84 (2.66)

Notes. N = number of participants; M = mean; SD = standard deviation; % = percent of the sample; IQR = interquartile range; CESD = Center for Epidemiologic Studies for Depression Scale; STAI = Spielberger State-Trait Anxiety Index

**Table 2.** Associations of endogenous estradiol with left and right hippocampal functional connections during verbal encoding that vary by *APOE4* carrier status.

Brain region	Brodmann area (BA)	Peak MNI coordinates (x,y,z)	t-score	Size (mm^3^)	Simple Effects β(SE)	
**Left Hippocampus**					***APOE4* Carriers**	***APOE4* Non-carriers**
L Inferior Temporal Lobe	20	−42, −14, −30	−3.96	52	−0.254 (0.104)*	0.150 (0.059)*
L Superior Parietal	7	−22, −64, 48	−3.68	56	−0.394 (0.244)	0.031 (0.153)
R Middle Frontal Gyrus/ Inferior Frontal		42, 22, 26	−3.58	170	−0.307 (0.139)*	0.058 (0.100)
R Fusiform		38, −8, −36	−3.45	27	−0.236 (0.137)	0.348 (0.106)**
R Fusiform		38, −72, −16	−3.41	45	−0.253 (0.179)	0.370 (0.098)***
L Middle Frontal Gyrus	6	−38, 12, 56	−3.39	37	−0.896 (0.290)**	0.146 (0.178)
L Amygdala/ PHG		−24, 2, −28	−3.32	29	−0.563 (0.195 **	0.290 (0.152)
L Lingual Gyrus		−24, −42, −2	−3.23	28	−0.098 (0.146)	0.110 (0.088)
R Superior Occipital		24, −72, 44	−3.03	26	−0.643 (0.340)	0.079 (0.183)
**Right Hippocampus**						
R Cingulate Gyrus		8, −2, 40	3.87	27	0.555 (0.133)***	−0.063 (0.104)
R Middle Frontal Gyrus	6	4, −20, 70	3.64	122	0.450 (0.140)**	−0.168 (0.118)
L Middle Frontal Gyrus	6	−24, 0, 58	3.55	35	0.365 (0.167)*	−0.146 (0.100)
L Superior Temporal Gyrus	38	−46, 16, −36	3.54	25	0.279 (0.214)	−0.009 (0.092)
R Superior Temporal Gyrus	22	52, 4, 4	3.5	145	0.858 (0.196)***	−0.250 (0.172)
L Precentral Gyrus	4	−58, −18, 44	3.45	44	0.445 (0.252)	−0.128 (0.126)
L Superior Temporal Gyrus	22	−54, 0, 2	3.3	99	0.644 (0.205)**	−0.208 (0.153)
L Amygdala/ PHG		−22, 2, −24	−3.59	41	−0.671 (0.253)*	0.287 (0.168)

Note: Abbreviations: (L) left, (R) right hemisphere, (SE) standard error. All fMRI results are cluster-size corrected at p<.05.

+ p<.10,

* p<.05,

** p<.01,

*** p<.001

**Table 3.** Associations of endogenous estradiol with left and right hippocampal functional connections during recognition that vary by *APOE4* carrier status.

Brain region	Brodmann area (BA)	Peak MNI coordinates (x,y,z)	t-score	Size (mm^3^)	Simple Effects β(SE)	

**Left Hippocampus**					***APOE4* Carriers**	***APOE4* Non-carriers**
L Inferior Parietal Lobe	40	−38, −42, 50	3.67	84	0.489 (0.239)*	−0.059 (0.134)
R Middle Frontal Gyrus	10	44, 42, 18	3.28	83	0.583 (0.185)**	−0.164 (0.139)
L Middle Frontal Gyrus		−40, 36, 30	3.17	25	0.477 (0.196)*	−0.216 (0.139)
**Right Hippocampus**						
R Middle Frontal Gyrus/ Inferior	46, 10	48, 44, 18	4.80	547		
Frontal Gyrus					0.455 (0.201)*	−0.117 (0.116)
L Inferior Parietal Lobe	40	−46, −40, 44	4.69	511	0.522 (0.231)*	−0.156 (0.133)
L Inferior Frontal Gyrus	9, 8	−52, 14, 40	4.15	118	0.780 (0.256)**	−0.200 (0.155)
L Middle Frontal Gyrus	46, 10	−50, 38, 18	4.00	179	0.536 (0.185)**	−0.158 (0.124)
L Middle Frontal Gyrus	6	−38, 10, 60	3.96	44	0.656 (0.249)*	−0.188 (0.123)
R Inferior Parietal Lobe	40	46, −42, 52	3.70	197	0.249 (0.266)	−0.081 (0.181)
R Middle Frontal Gyrus	6	32, 14, 62	3.62	44	0.586 (0.203)**	−0.182 (0.131)
R Superior Frontal Gyrus	6	20, 18, 66	3.53	41	0.551 (0.174)**	−0.211 (0.130)
R Cingulate Gyrus	23	4, −24, 34	3.52	69	0.933 (0.262)**	0.066 (0.174)
R Inferior Temporal Gyrus	20	52, −2, −34	3.45	39	0.378 (0.216)	0.057 (0.156)
R Inferior Parietal Lobe	7, 40	40, −60, 46	3.42	152	0.497 (0.149)**	0.044 (0.104)
R Middle Temporal Gyrus	21	66, −42, −10	3.37	64	0.501 (0.180)**	0.059 (0.154)
R Precuneus		8, −66, 46	3.34	29	0.882 (0.366)*	0.091 (0.204)
L Precuneus	7	−8, −66, 48	3.23	34	0.774 (0.257)**	−0.264 (0.195)
R Inferior Parietal Lobe	40	60, −34, 42	3.10	53	0.456 (0.329)	−0.127 (0.182)
L Hippocampus/ PHG		−22, −20, −14	−3.01	29	−0.470 (0.221)*	0.471 (0.151)**

Abbreviations: (L) left, (R) right hemisphere.

Note: All fMRI results are cluster-size corrected at p<.05.

+ p<.10,

* p<.05,

** p<.01

**Table 4.** Summarization of associations with endogenous estradiol levels in hippocampal functional connections during verbal encoding that vary by *APOE4* carrier status and influence of Alzheimer’s biomarkers and cognition.

Brain region	Relationship to Estradiol		AD Biomarkers		Cognition	
	*APOE4* Carriers	*APOE4* Non-carriers	*APOE4* Carriers	*APOE4* Non-carriers	*APOE4* Carriers	*APOE4* Non-carriers

**Left Hippocampus**						
L Inferior Temporal Lobe	− *	+ *		+ pTau231*		
L Superior Parietal						
R Middle Frontal Gyrus/Inferior Frontal	− *	+ **				
R Fusiform (c27)		+ ***				
R Fusiform (c45)	− **		− pTau181* − pTau231**			
L Middle Frontal Gyrus	− *		− pTau231*			
L Amygdala/ PHG	− **		+ Aβ42/40*			
L Lingual Gyrus						
R Superior Occipital						
**Right Hippocampus**						
R Cingulate Gyrus	+ ***		− Aβ42/40*			+ Long Delay Recall*
R Middle Frontal Gyrus	+ **					+ CVLT Semantic Clustering*
						+ Long Delay Recall*
L Middle Frontal Gyrus	+ *					
L Superior Temporal						+ CVLT Semantic
Gyrus (c25)						Clustering**
R Superior Temporal Gyrus	+ **		− pTau181*			+ CVLT Verbal Learning* + CVLT Semantic Clustering** + Short Delay Recall*
L Precentral Gyrus						+ Long Delay Recall* + CVLT Semantic Clustering*
L Superior Temporal	+ ***					
Gyrus (c99)						
L Amygdala/ PHG	− *					

Abbreviations: (AD) Alzheimer’s disease, (L) left, (R) right hemisphere, (c) cluster size for regions with multiple significant areas.

(+) positive association, (−) negative association

* p<.05,

** p<.01,

*** p<.001

**Table 5.** Summarization of associations with endogenous estradiol levels in hippocampal functional connections during verbal recognition that vary by *APOE4* carrier status and influence of Alzheimer’s biomarkers and cognition.

Brain region	Relationship to Estradiol		AD Biomarkers		Cognition	
	*APOE4* Carriers	*APOE4* Non-carriers	*APOE4* Carriers	*APOE4* Non-carriers	*APOE4* Carriers	*APOE4* Non-carriers

**Left Hippocampus**						
L Inferior Parietal Lobe	+ *				+ Short Delay Recall*	
R Middle Frontal Gyrus	+ **					
L Middle Frontal Gyrus	+ *					
**Right Hippocampus**						
R Middle Frontal Gyrus/Inferior Frontal Gyrus (c547)	+*					
L Inferior Parietal Lobe	+ *			+ pTau181*		
L Inferior Frontal Gyrus	+ **					
L Middle Frontal Gyrus (c179)	+ **		− Aβ42/40*			
L Middle Frontal Gyrus (c44)	+ *		− Aβ42/40*			
R Inferior Parietal Lobe (c197)			− Aβ42/40**			
R Middle Frontal Gyrus (c44)	+ **					
R Superior Frontal Gyrus			− Aβ42/40*		CVLT Verbal Learning* − CVLT Semantic Clustering* − Short Delay Recall*	
R Cingulate Gyrus	+ **					
R Inferior Temporal Gyrus	+ **				− CVLT Verbal Learning*	
R Inferior Parietal Lobe (c152)						
R Middle Temporal Gyrus	+ **					
R Precuneus	+ *			+ pTau181*		
L Precuneus	+ **					
R Inferior Parietal Lobe (c53)						
L Hippocampus/ PHG	− *	+ **				

Abbreviations: (AD) Alzheimer’s disease, (L) left, (R) right hemisphere, (c) cluster size for regions with multiple significant areas.

(+) positive association, (−) negative association

* p<.05,

** p<.01,

*** p<.001
