# Supplementary material for: Estradiol and memory circuitry in the postmenopause: Modification by APOE4
Source: Res Sq. 2026 Aug 4:rs.3.rs-10057599. Preprint. [Version 1] doi: 10.21203/rs.3.rs-10057599/v1 (PMC13484806; doi:10.21203/rs.3.rs-10057599/v1)
Supplement: Supplementary file 1 [file NIHPPrs10057599v1-supplement-1.pdf]

Supplemental Tables

Brain region	pTau 181 β(SE)		pTau 231 β(SE)		Aβ42/40 β(SE)	
**Left Hippocampus**						
L Amygdala	0.006	(0.005)	0.003	(0.003)	11.269*	(5.478)
L Inferior Temporal Lobe	0.002	(0.002)	0.000	(0.002)	4.405	(2.874)
L Lingual	0.002	(0.003)	0.001	(0.002)	-3.006	(3.172)
L Middle Frontal Gyrus (c37)	-0.005+	(0.003)	-0.005*	(0.002)	0.433	(5.207)
L Superior Parietal	0.003	(0.005)	0.001	(0.003)	4.417	(5.491)
R Fusiform (c27)	0.000	(0.004)	0.000	(0.003)	4.438	(4.591)
R Fusiform (c45)	-0.015*	(0.007)	-0.013**	(0.005)	-4.882	(8.733)
R Middle Frontal Gyrus (c170)	-0.006+	(0.003)	-0.004+	(0.002)	-0.224	(4.401)
R Superior Occipital	0.000	(0.007)	-0.002	(0.005)	-1.453	(8.833)
**Right Hippocampus**						
L Amygdala	0.011+	(0.006)	0.006	(0.004)	13.915+	(7.044)
L Middle Frontal Gyrus	-0.003	(0.004)	-0.001	(0.003)	4.425	(4.577)
L Precentral Gyrus	-0.002	(0.006)	0.000	(0.004)	-1.552	(6.586)
L Superior Temporal Gyrus (c99)	-0.009+	(0.005)	-0.005	(0.004)	-10.924+	(6.235)
L Superior Temporal Gyrus (c25)	0.004	(0.004)	0.003	(0.003)	4.083	(4.691)
R Cingulate Gyrus	-0.006+	(0.003)	-0.004	(0.002)	-9.356*	(4.010)
R Middle Frontal Gyrus	-0.006+	(0.003)	-0.004	(0.002)	-3.375	(4.121)
R Superior Temporal Gyrus	-0.010*	(0.005)	-0.006	(0.004)	-7.554	(6.003)

Supplemental Table 1. Results from verbal encoding task in *APOE4* carriers and associations with Alzheimer's disease biomarkers.
Associations between endogenous estradiol and left and right hippocampal functional connectivity in *APOE4* carriers with blood-based AD biomarkers during verbal encoding.
Abbreviations: (L) left, (R) right hemisphere, (c) cluster size for regions with multiple significant areas.
+ p<.10, * p<.05, ** p<.01

**Brain region**	**pTau 181** **β(SE)**		**pTau 231** **β(SE)**		**Aβ42/40** **β(SE)**	
**Left Hippocampus**						
L Amygdala	-0.002	(0.003)	-0.002	(0.002)	1.737	(2.976)
L Inferior Temporal Lobe	0.001	(0.001)	0.002*	(0.001)	1.755	(1.183)
L Lingual	0.003+	(0.002)	0.003+	(0.001)	-0.667	(1.787)
L Middle Frontal Gyrus (c37)	-0.004	(0.002)	-0.001	(0.002)	2.648	(2.168)
L Superior Parietal	0.006	(0.004)	0.004+	(0.002)	1.758	(3.046)
R Fusiform (c27)	0.002	(0.002)	0.001	(0.002)	0.517	(2.030)
R Fusiform (c45)	0.004	(0.004)	0.002	(0.003)	6.247+	(3.491)
R Middle Frontal Gyrus (c170)	0.002	(0.002)	0.003+	(0.002)	3.536+	(1.964)
R Superior Occipital	0.002	(0.004)	0.003	(0.003)	2.372	(3.588)
**Right Hippocampus**						
L Amygdala	0.000	(0.003)	-0.003	(0.003)	1.895	(3.491)
L Middle Frontal Gyrus	0.000	(0.002)	0.000	(0.002)	2.672	(2.025)
L Precentral Gyrus	-0.002	(0.003)	-0.001	(0.002)	-0.688	(2.641)
L Superior Temporal Gyrus (c99)	0.003	(0.004)	-0.001	(0.003)	-4.401	(3.648)
L Superior Temporal Gyrus (c25)	0.000	(0.002)	-0.001	(0.001)	-2.186	(1.825)
R Cingulate Gyrus	0.000	(0.002)	0.000	(0.002)	0.032	(2.081)
R Middle Frontal Gyrus	0.000	(0.002)	0.000	(0.002)	-0.225	(2.377)
R Superior Temporal Gyrus	0.000	(0.003)	-0.003	(0.002)	-3.890	(3.074)

Supplemental Table 2. Results from verbal encoding task in *APOE4* non-carriers and associations with Alzheimer's disease biomarkers**.**
Associations between endogenous estradiol and left and right hippocampal functional connectivity in *APOE4* non-carriers with blood-based AD biomarkers during verbal encoding. Abbreviations: (L) left, (R) right hemisphere, (c) cluster size for regions with multiple significant areas.
+ p<.10, * p<.05, ** p<.01, *** p<.001

Brain region	pTau 181 β(SE)		pTau 231 β(SE)		Aβ42/40 β(SE)	
**Left Hippocampus**						
L Inferior Parietal Lobe	-0.003	(0.004)	-0.001	(0.003)	-4.459	(5.368)
L Middle Frontal Gyrus	-0.002	(0.005)	-0.001	(0.003)	-6.745	(5.493)
R Middle Frontal Gyrus	-0.005	(0.004)	-0.003	(0.003)	-9.277+	(5.074)
**Right Hippocampus**						
L Hippocampus	0.008	(0.005)	0.003	(0.004)	6.347	(5.883)
L Inferior Frontal Gyrus	-0.003	(0.005)	-0.002	(0.004)	-10.435+	(5.950)
L Inferior Parietal Lobe (c511)	-0.006	(0.004)	-0.004	(0.003)	-8.549	(5.169)
L Middle Frontal Gyrus (c179)	-0.004	(0.004)	-0.003	(0.003)	-8.943*	(4.245)
L Middle Frontal Gyrus (c44)	-0.004	(0.004)	-0.003	(0.003)	-9.104	(5.747)
L Precuneus	0.004	(0.006)	0.005	(0.004)	3.542	(8.568)
R Cingulate	0.007	(0.007)	0.007	(0.005)	3.486	(8.131)
R Inferior Parietal Lobe (c152)	0.002	(0.006)	0.000	(0.005)	-7.200	(7.285)
R Inferior Parietal Lobe (c197)	0.000	(0.004)	0.000	(0.003)	-13.439**	(4.617)
R Inferior Parietal Lobe (c53)	-0.007	(0.004)	-0.006+	(0.003)	-3.515	(5.417)
R Inferior Temporal Gyrus	0.000	(0.004)	0.001	(0.003)	-3.174	(4.532)
R Middle Temporal Gyrus	0.002	(0.004)	0.002	(0.003)	4.271	(4.267)
R Middle Frontal Gyrus (c44)	-0.004	(0.004)	-0.002	(0.003)	-11.642*	(4.514)
R Middle Frontal Gyrus (c547)	-0.005	(0.004)	-0.002	(0.003)	-8.159+	(4.128)
R Precuneus	-0.006	(0.007)	-0.004	(0.005)	-6.986	(8.643)
R Superior Frontal Gyrus	-0.001	(0.004)	-0.001	(0.003)	-10.943*	(4.616)

Supplemental Table 3. Results from verbal recognition task in *APOE4* carriers and associations with Alzheimer's disease biomarkers**.**

Associations between endogenous estradiol and left and right hippocampal functional connectivity in *APOE4* carriers with blood-based AD biomarkers during verbal recognition.

Abbreviations: (L) left, (R) right hemisphere, (c) cluster size for regions with multiple significant areas.

+ p<.10, * p<.05, ** p<.01

Brain region	pTau 181 β(SE)		pTau 231 β(SE)		Aβ42/40 β(SE)	
**Left Hippocampus**						
L Inferior Parietal Lobe	0.006+	(0.003)	0.003	(0.002)	2.350	(2.726)
L Middle Frontal Gyrus	0.005+	(0.003)	0.002	(0.002)	-1.145	(2.857)
R Middle Frontal Gyrus	0.003	(0.003)	-0.001	(0.002)	-2.693	(2.743)
**Right Hippocampus**						
L Hippocampus	0.002	(0.003)	0.001	(0.002)	-1.312	(3.096)
L Inferior Frontal Gyrus	0.002	(0.003)	0.001	(0.002)	-1.559	(3.124)
L Inferior Parietal Lobe (c511)	0.007*	(0.003)	0.002	(0.002)	0.900	(2.656)
L Middle Frontal Gyrus (c179)	0.003	(0.003)	0.000	(0.002)	2.634	(2.498)
L Middle Frontal Gyrus (c44)	0.003	(0.002)	0.002	(0.002)	-3.407	(2.410)
L Precuneus	0.004	(0.004)	0.003	(0.003)	7.029+	(3.856)
R Cingulate	0.000	(0.003)	0.003	(0.003)	5.034	(3.419)
R Inferior Parietal Lobe (c152)	0.006	(0.004)	0.000	(0.003)	3.460	(3.648)
R Inferior Parietal Lobe (c197)	0.000	(0.004)	0.000	(0.003)	-2.654	(3.654)
R Inferior Parietal Lobe (c53)	0.003	(0.003)	0.001	(0.002)	-0.378	(3.095)
R Inferior Temporal Gyrus	0.001	(0.002)	0.001	(0.002)	1.857	(2.023)
R Middle Temporal Gyrus	0.002	(0.003)	-0.001	(0.002)	-2.045	(3.082)
R Middle Frontal Gyrus (c44)	0.001	(0.003)	0.000	(0.002)	-3.488	(2.642)
R Middle Frontal Gyrus (c547)	0.002	(0.002)	0.000	(0.002)	-3.260	(2.323)
R Precuneus	0.009*	(0.004)	0.004	(0.003)	5.486	(3.997)
R Superior Frontal Gyrus	0.000	(0.003)	0.000	(0.002)	-2.963	(2.623)

Supplemental Table 4. Results from verbal recognition task in *APOE4* non-carriers and associations with Alzheimer's disease biomarkers.
Associations between endogenous estradiol and left and right hippocampal functional connectivity in *APOE4* non-carriers with blood-based AD biomarkers during verbal recognition.
Abbreviations: (L) left, (R) right hemisphere, (c) cluster size for regions with multiple significant areas.
+ $p<.10$, * $p<.05$, ** $p<.01$

Brain Region	MOCA β(SE)		CVLT Verbal Learning β(SE)		CVLT Semantic Clustering β(SE)		Short Delay Recall β(SE)		Long Delay Recall β(SE)		Task Accuracy β(SE)		Task Verbal Learning β(SE)		Task Semantic Clustering β(SE)	
**Left Hippocampus**																
L Amygdala	0.007	(0.031)	0.008	(0.010)	0.032	(0.089)	0.016	(0.029)	0.023	(0.033)	0.913	(0.766)	0.000	(0.014)	-0.005	(0.018)
L Inferior Temporal Lobe	0.020	(0.015)	0.004	(0.005)	-0.023	(0.045)	0.012	(0.015)	0.018	(0.017)	0.154	(0.402)	-0.002	(0.007)	-0.005	(0.009)
L Lingual	-0.018	(0.017)	0.002	(0.005)	-0.029	(0.050)	0.002	(0.017)	-0.018	(0.019)	-0.113	(0.450)	-0.006	(0.007)	-0.009	(0.009)
L Middle Frontal Gyrus (c37)	0.000	(0.023)	0.004	(0.006)	0.008	(0.058)	0.003	(0.019)	0.026	(0.021)	-0.152	(0.511)	0.002	(0.009)	0.001	(0.012)
L Superior Parietal	-0.058*	(0.028)	-0.003	(0.009)	-0.129	(0.081)	-0.012	(0.029)	-0.015	(0.033)	-0.100	(0.777)	-0.012	(0.013)	-0.018	(0.016)
R Fusiform (c27)	0.000	(0.025)	0.008	(0.008)	-0.050	(0.069)	0.035	(0.022)	0.009	(0.026)	0.782	(0.609)	0.007	(0.011)	0.005	(0.014)
R Fusiform (c45)	-0.070	(0.046)	0.006	(0.015)	0.155	(0.131)	-0.004	(0.044)	0.040	(0.049)	0.328	(1.248)	-0.020	(0.021)	-0.023	(0.026)
R Middle Frontal Gyrus (c170)	-0.009	(0.023)	-0.002	(0.007)	-0.037	(0.057)	0.001	(0.019)	0.013	(0.022)	0.364	(0.511)	0.004	(0.009)	0.000	(0.011)
R Superior Occipital	-0.058	(0.042)	0.008	(0.014)	-0.045	(0.121)	-0.029	(0.043)	0.003	(0.049)	0.824	(1.113)	0.020	(0.018)	0.013	(0.023)
**Right Hippocampus**																
L Amygdala	-0.030	(0.039)	0.007	(0.012)	0.024	(0.110)	0.007	(0.036)	-0.001	(0.041)	0.614	(0.995)	-0.011	(0.018)	-0.019	(0.023)
L Middle Frontal Gyrus	0.005	(0.025)	-0.008	(0.008)	0.016	(0.073)	0.004	(0.024)	-0.009	(0.027)	-0.571	(0.636)	-0.010	(0.011)	-0.020	(0.014)
L Precentral Gyrus	-0.018	(0.035)	-0.006	(0.011)	0.047	(0.099)	-0.008	(0.033)	-0.020	(0.037)	-0.431	(0.889)	-0.004	(0.016)	-0.022	(0.019)
L Superior Temporal Gyrus (c99)	-0.028	(0.034)	-0.006	(0.011)	0.025	(0.098)	-0.017	(0.032)	-0.028	(0.036)	-0.445	(0.869)	0.005	(0.015)	-0.004	(0.019)
L Superior Temporal Gyrus (c25)	0.014	(0.026)	0.007	(0.007)	0.051	(0.069)	0.024	(0.024)	0.026	(0.027)	-0.066	(0.673)	-0.002	(0.011)	-0.006	(0.014)
R Cingulate Gyrus	-0.033	(0.022)	0.003	(0.007)	0.011	(0.065)	0.000	(0.021)	-0.015	(0.024)	-0.131	(0.583)	0.009	(0.010)	0.001	(0.013)
R Middle Frontal Gyrus	0.008	(0.022)	-0.009	(0.007)	0.040	(0.061)	-0.016	(0.021)	-0.008	(0.023)	-0.523	(0.569)	0.006	(0.010)	-0.002	(0.013)
R Superior Temporal Gyrus	-0.029	(0.032)	-0.007	(0.010)	0.102	(0.090)	-0.007	(0.030)	-0.018	(0.034)	-0.007	(0.830)	0.011	(0.014)	0.005	(0.018)

Supplemental Table 5. Results from verbal encoding task in *APOE4* carriers and associations with measures of global and verbal memory performance.

Associations between endogenous estradiol and left and right hippocampal functional connectivity in *APOE4* carriers with measures of cognition during verbal encoding. Abbreviations: (L) left, (R) right hemisphere, (c) cluster size for regions with multiple significant areas.

+ p<.10, * p<.05, ** p<.01

Brain Region	MOCA β(SE)		CVLT Verbal Learning β(SE)		CVLT Semantic Clustering β(SE)		Short Delay Recall β(SE)		Long Delay Recall β(SE)		Task Accuracy β(SE)		Task Verbal Learning β(SE)		Task Semantic Clustering β(SE)	
**Left Hippocampus**																
L Amygdala	0.033+	(0.017)	0.002	(0.005)	0.027	(0.048)	-0.004	(0.017)	0.000	(0.017)	0.230	(0.468)	0.004	(0.008)	0.005	(0.011)
L Inferior Temporal Lobe	-0.008	(0.007)	0.001	(0.002)	0.014	(0.019)	0.006	(0.006)	0.006	(0.007)	-0.273	(0.180)	-0.002	(0.003)	-0.006	(0.004)
L Lingual	-0.004	(0.010)	0.001	(0.003)	0.024	(0.027)	0.011	(0.009)	0.014	(0.010)	0.263	(0.273)	0.003	(0.005)	-0.001	(0.006)
L Middle Frontal Gyrus (c37)	0.006	(0.013)	0.005	(0.004)	0.062+	(0.034)	0.019	(0.012)	0.019	(0.012)	0.087	(0.344)	-0.001	(0.006)	-0.002	(0.008)
L Superior Parietal	-0.004	(0.018)	0.002	(0.005)	0.008	(0.048)	0.011	(0.017)	0.016	(0.017)	0.293	(0.472)	0.009	(0.008)	0.002	(0.011)
R Fusiform (c27)	-0.002	(0.012)	0.001	(0.004)	0.063+	(0.032)	0.017	(0.011)	0.014	(0.011)	-0.343	(0.312)	0.002	(0.006)	-0.002	(0.007)
R Fusiform (c45)	0.006	(0.021)	0.002	(0.006)	0.040	(0.057)	0.016	(0.019)	0.010	(0.020)	-0.020	(0.556)	0.014	(0.009)	0.014	(0.013)
R Middle Frontal Gyrus (c170)	0.017	(0.012)	-0.001	(0.003)	0.051	(0.031)	0.017	(0.011)	0.016	(0.011)	-0.075	(0.311)	0.003	(0.005)	-0.001	(0.007)
R Superior Occipital	0.018	(0.021)	0.007	(0.006)	0.062	(0.056)	0.023	(0.019)	0.031	(0.020)	0.859	(0.525)	0.024*	(0.009)	0.018	(0.012)
**Right Hippocampus**																
L Amygdala	0.026	(0.019)	0.000	(0.006)	0.005	(0.052)	-0.018	(0.018)	-0.006	(0.018)	0.070	(0.508)	0.014	(0.009)	0.015	(0.011)
L Middle Frontal Gyrus	0.007	(0.012)	0.003	(0.003)	0.035	(0.031)	0.002	(0.011)	0.010	(0.011)	0.410	(0.302)	0.014**	(0.005)	0.013+	(0.007)
L Precentral Gyrus	0.021	(0.014)	0.006	(0.004)	0.096*	(0.039)	0.020	(0.014)	0.020	(0.014)	0.687+	(0.371)	0.022**	(0.006)	0.020*	(0.009)
L Superior Temporal Gyrus (c99)	0.044*	(0.020)	0.004	(0.006)	0.058	(0.055)	0.024	(0.019)	0.032	(0.020)	0.803	(0.534)	0.026**	(0.009)	0.027*	(0.012)
L Superior Temporal Gyrus (c25)	-0.008	(0.011)	-0.006+	(0.003)	-0.047	(0.029)	-0.013	(0.010)	-0.009	(0.010)	-0.254	(0.278)	-0.003	(0.005)	-0.009	(0.006)
R Cingulate Gyrus	0.014	(0.012)	0.005	(0.003)	0.083**	(0.031)	0.018+	(0.011)	0.027*	(0.011)	0.386	(0.309)	0.011+	(0.006)	0.007	(0.007)
R Middle Frontal Gyrus	0.027+	(0.014)	0.007+	(0.004)	0.088*	(0.036)	0.018	(0.013)	0.033*	(0.013)	0.620+	(0.348)	0.019**	(0.006)	0.020*	(0.008)
R Superior Temporal Gyrus	0.052**	(0.017)	0.013*	(0.005)	0.126**	(0.047)	0.038*	(0.016)	0.047**	(0.017)	0.619	(0.454)	0.030***	(0.008)	0.032**	(0.010)

Supplemental Table 6. Results from verbal encoding task in *APOE4* non-carriers and associations with measures of global and verbal memory performance.

Associations between endogenous estradiol and left and right hippocampal functional connectivity in *APOE4* non-carriers with measures of cognition during verbal encoding. Abbreviations: (L) left, (R) right hemisphere, (c) cluster size for regions with multiple significant areas.

+ p<.10, * p<.05, ** p<.01

Brain Region	MOCA β(SE)		CVLT Verbal Learning β(SE)		CVLT Semantic Clustering β(SE)		Short Delay Recall β(SE)		Long Delay Recall β(SE)		Task Accuracy β(SE)		Task Verbal Learning β(SE)		Task Semantic Clustering β(SE)	
**Left Hippocampus**																
L Inferior Parietal Lobe	0.012	(0.030)	0.011	(0.009)	0.082	(0.085)	0.058*	(0.028)	0.057+	(0.032)	0.702	(0.769)	0.019	(0.012)	0.021	(0.016)
L Middle Frontal Gyrus	0.012	(0.030)	0.004	(0.009)	0.047	(0.085)	0.039	(0.027)	0.024	(0.031)	0.428	(0.756)	0.006	(0.013)	0.002	(0.017)
R Middle Frontal Gyrus	0.027	(0.029)	-0.006	(0.009)	-0.049	(0.084)	-0.001	(0.028)	-0.011	(0.031)	-0.245	(0.748)	0.000	(0.013)	-0.006	(0.017)
**Right Hippocampus**																
L Hippocampus	-0.002	(0.033)	-0.006	(0.010)	0.103	(0.094)	0.001	(0.031)	0.005	(0.035)	-0.443	(0.857)	-0.006	(0.015)	0.005	(0.019)
L Inferior Frontal Gyrus	0.022	(0.034)	-0.008	(0.011)	-0.125	(0.097)	-0.021	(0.034)	-0.025	(0.038)	-0.646	(0.914)	0.011	(0.015)	0.004	(0.019)
L Inferior Parietal Lobe (c511)	0.010	(0.029)	0.003	(0.009)	0.031	(0.085)	0.032	(0.029)	0.029	(0.033)	-0.268	(0.761)	0.010	(0.012)	0.011	(0.016)
L Middle Frontal Gyrus (c179)	0.006	(0.024)	-0.013+	(0.007)	-0.051	(0.069)	-0.011	(0.024)	-0.026	(0.027)	-0.037	(0.634)	-0.001	(0.010)	-0.006	(0.013)
L Middle Frontal Gyrus (c44)	0.032	(0.030)	-0.007	(0.009)	-0.060	(0.088)	-0.005	(0.030)	-0.017	(0.033)	0.648	(0.771)	0.017	(0.013)	0.017	(0.016)
L Precuneus	-0.001	(0.041)	0.006	(0.013)	0.024	(0.116)	0.014	(0.038)	0.011	(0.044)	0.146	(1.028)	0.006	(0.018)	0.006	(0.023)
R Cingulate	0.056	(0.042)	0.001	(0.013)	0.060	(0.113)	0.016	(0.037)	0.027	(0.042)	-0.604	(1.091)	0.009	(0.019)	0.014	(0.024)
R Inferior Parietal Lobe (c152)	0.030	(0.040)	-0.003	(0.013)	0.050	(0.115)	-0.021	(0.039)	0.019	(0.045)	0.296	(1.056)	0.014	(0.017)	0.011	(0.022)
R Inferior Parietal Lobe (c197)	-0.007	(0.030)	-0.013	(0.009)	-0.074	(0.081)	-0.048+	(0.027)	-0.028	(0.032)	-0.417	(0.766)	-0.007	(0.012)	-0.013	(0.016)
R Inferior Parietal Lobe (c53)	-0.026	(0.030)	-0.008	(0.009)	-0.083	(0.085)	-0.034	(0.028)	-0.044	(0.031)	-0.906	(0.750)	-0.005	(0.013)	-0.012	(0.017)
R Inferior Temporal Gyrus	-0.024	(0.024)	-0.015*	(0.007)	0.008	(0.069)	-0.023	(0.022)	-0.023	(0.025)	-0.625	(0.603)	-0.012	(0.010)	-0.016	(0.013)
R Middle Temporal Gyrus	0.056*	(0.022)	0.003	(0.007)	0.114+	(0.065)	0.030	(0.023)	0.033	(0.026)	0.781	(0.600)	0.025*	(0.009)	0.025*	(0.012)
R Middle Frontal Gyrus (c44)	0.006	(0.027)	-0.009	(0.008)	-0.097	(0.075)	-0.010	(0.026)	-0.040	(0.030)	-0.694	(0.686)	-0.007	(0.011)	-0.009	(0.015)
R Middle Frontal Gyrus (c547)	0.004	(0.024)	-0.005	(0.008)	-0.007	(0.069)	0.012	(0.024)	-0.008	(0.027)	-0.467	(0.617)	-0.011	(0.010)	-0.010	(0.013)
R Precuneus	0.028	(0.047)	0.003	(0.015)	0.058	(0.135)	0.026	(0.046)	0.031	(0.053)	0.163	(1.239)	0.007	(0.020)	0.006	(0.026)
R Superior Frontal Gyrus	0.007	(0.027)	-0.016*	(0.008)	-0.152*	(0.069)	-0.049*	(0.023)	-0.041	(0.026)	-1.085	(0.645)	-0.012	(0.011)	-0.023	(0.014)

Supplemental Table 7. Results from verbal recognition task in *APOE4* carriers and associations with measures of global and verbal memory performance.

Associations between endogenous estradiol and left and right hippocampal functional connectivity in *APOE4* carriers with measures of cognition during verbal recognition. Abbreviations: (L) left, (R) right hemisphere, (c) cluster size for regions with multiple significant areas.

+ $p<.10$, * $p<.05$, ** $p<.01$

Brain Region	MOCA β(SE)		CVLT Verbal Learning β(SE)		CVLT Semantic Clustering β(SE)		Short Delay Recall β(SE)		Long Delay Recall β(SE)		Task Accuracy β(SE)		Task Verbal Learning β(SE)		Task Semantic Clustering β(SE)	
**Left Hippocampus**																
L Inferior Parietal Lobe	0.024	(0.015)	0.000	(0.005)	-0.001	(0.042)	-0.012	(0.015)	-0.005	(0.015)	-0.036	(0.414)	0.001	(0.007)	0.006	(0.009)
L Middle Frontal Gyrus	0.018	(0.016)	0.002	(0.005)	-0.023	(0.044)	0.002	(0.015)	0.003	(0.016)	0.093	(0.435)	-0.003	(0.007)	0.001	(0.010)
R Middle Frontal Gyrus	0.009	(0.016)	-0.001	(0.005)	-0.037	(0.043)	-0.020	(0.015)	-0.010	(0.015)	0.194	(0.419)	-0.001	(0.007)	0.002	(0.010)
**Right Hippocampus**																
L Hippocampus	-0.025	(0.018)	-0.002	(0.005)	-0.034	(0.050)	-0.007	(0.017)	-0.022	(0.017)	0.605	(0.472)	0.004	(0.008)	0.005	(0.011)
L Inferior Frontal Gyrus	0.007	(0.018)	0.002	(0.005)	0.022	(0.049)	0.002	(0.017)	-0.004	(0.017)	-0.435	(0.473)	0.002	(0.008)	0.004	(0.011)
L Inferior Parietal Lobe (c511)	0.019	(0.015)	-0.003	(0.005)	-0.018	(0.043)	-0.024	(0.014)	-0.019	(0.015)	-0.098	(0.414)	0.003	(0.007)	0.008	(0.010)
L Middle Frontal Gyrus (c179)	0.009	(0.014)	-0.001	(0.004)	0.011	(0.040)	-0.012	(0.014)	-0.007	(0.014)	-0.292	(0.382)	-0.001	(0.007)	0.006	(0.009)
L Middle Frontal Gyrus (c44)	-0.003	(0.014)	-0.001	(0.004)	-0.007	(0.039)	-0.002	(0.013)	0.001	(0.014)	-0.370	(0.373)	-0.001	(0.007)	-0.002	(0.009)
L Precuneus	0.000	(0.023)	0.000	(0.007)	0.001	(0.062)	-0.024	(0.021)	-0.008	(0.022)	-0.515	(0.600)	0.000	(0.010)	0.017	(0.014)
R Cingulate	0.025	(0.020)	0.001	(0.006)	0.041	(0.053)	0.008	(0.018)	0.006	(0.019)	0.103	(0.525)	-0.004	(0.009)	0.002	(0.012)
R Inferior Parietal Lobe (c152)	0.021	(0.021)	-0.005	(0.006)	-0.040	(0.059)	-0.033+	(0.020)	-0.031	(0.021)	0.153	(0.563)	0.011	(0.010)	0.020	(0.013)
R Inferior Parietal Lobe (c197)	0.020	(0.021)	-0.004	(0.006)	-0.052	(0.058)	-0.037+	(0.020)	-0.036+	(0.020)	-0.107	(0.555)	-0.006	(0.010)	-0.005	(0.013)
R Inferior Parietal Lobe (c53)	0.010	(0.018)	-0.003	(0.005)	-0.016	(0.048)	-0.015	(0.017)	-0.012	(0.017)	0.033	(0.472)	-0.005	(0.008)	-0.007	(0.011)
R Inferior Temporal Gyrus	-0.007	(0.012)	-0.002	(0.004)	-0.012	(0.032)	-0.007	(0.011)	-0.010	(0.011)	-0.064	(0.314)	-0.003	(0.005)	-0.005	(0.007)
R Middle Temporal Gyrus	0.012	(0.018)	0.002	(0.005)	0.007	(0.049)	-0.004	(0.017)	-0.016	(0.017)	-0.046	(0.468)	0.003	(0.008)	0.005	(0.011)
R Middle Frontal Gyrus (c44)	0.023	(0.015)	-0.007	(0.005)	-0.054	(0.042)	-0.020	(0.015)	-0.018	(0.015)	-0.012	(0.409)	-0.002	(0.007)	-0.002	(0.009)
R Middle Frontal Gyrus (c547)	0.012	(0.013)	-0.004	(0.004)	-0.043	(0.036)	-0.022+	(0.012)	-0.021+	(0.013)	-0.254	(0.353)	-0.006	(0.006)	-0.001	(0.008)
R Precuneus	0.004	(0.024)	0.003	(0.007)	-0.003	(0.064)	-0.011	(0.022)	-0.009	(0.023)	-0.132	(0.623)	-0.001	(0.011)	0.012	(0.014)
R Superior Frontal Gyrus	0.034*	(0.015)	0.003	(0.005)	0.051	(0.041)	0.004	(0.014)	0.009	(0.015)	0.331	(0.399)	0.011	(0.007)	0.018*	(0.009)

Supplemental Table 8. Results from verbal recognition task in *APOE4* non-carriers and associations with measures of global and verbal memory performance.

Associations between endogenous estradiol and left and right hippocampal functional connectivity in *APOE4* non-carriers with measures of cognition during verbal recognition. Abbreviations: (L) left, (R) right hemisphere, (c) cluster size for regions with multiple significant areas.

+ $p<.10$, * $p<.05$, ** $p<.01$
